# Massive right-sided Bochdalek hernia with two unusual findings: a case report

**DOI:** 10.1186/1752-1947-5-519

**Published:** 2011-10-21

**Authors:** Subrato J Deb

**Affiliations:** 1 Thoracic Surgical Services, Western Maryland Regional Medical Center, 12502 Willowbrook Road, Suite 470, Cumberland, Maryland 21502, USA

## Abstract

**Introduction:**

In this report, the case of an adult patient with a massive right-sided Bochdalek hernia with multiple displaced abdominal organs, including the liver and gallbladder, is described. This patient presented with acute cholecystitis of the malpositioned gallbladder. During surgery, nodular regenerative hyperplasia of the liver was also found. To the best of this author's knowledge, these two entities have never been reported in association with this rare condition.

**Case presentation:**

A 54-year-old Caucasian man presented with nausea and epigastric pain. He had a known history of right-sided Bochdalek hernia which was being managed expectantly. A computerized tomogram revealed the massive hernia with displaced stomach, liver, intestine and omentum into his right thorax. It was believed that our patient had bowel incarceration and he was therefore taken to surgery, where acute cholecystitis and a macronodular liver was identified. A thoracoabdominal approach was used to remove his gallbladder, reduce the herniated viscera and reconstruct his diaphragm. A liver biopsy identified nodular regenerative hyperplasia of the ectopic liver. There were no postoperative complications and at 12 month follow-up, our patient continues to do well.

**Conclusion:**

This case report describes two unusual findings associated with a congenital Bochdalek diaphragmatic hernia that have never been reported. In addition, unique caveats to the surgical management of this complex rare condition are discussed.

## Introduction

Diaphragmatic hernias of Bochdalek are rare congenital defects that occur along the posterolateral aspect of the diaphragm. In a recent review, the incidence of adult Bochdalek hernias was noted at 0.17% based on 13,138 abdominal computed tomography (CT) scans [1]. Less than 200 cases of such hernias have been described in adults to date [2]. Unlike infants who present with respiratory distress, adults with Bochdalek hernias typically present with complications of the herniated abdominal viscera, most commonly bowel obstruction. In this report, the case of a 54-year-old man presenting with acute cholecystitis of the ectopic intrathoracic gallbladder is described. In addition, our patient was noted to have a rare hepatic condition called nodular regenerative hyperplasia (NRH). The etiology of this liver abnormality is unknown but may be due to abnormalities in hepatic blood flow.

## Case presentation

**Figure 1:**
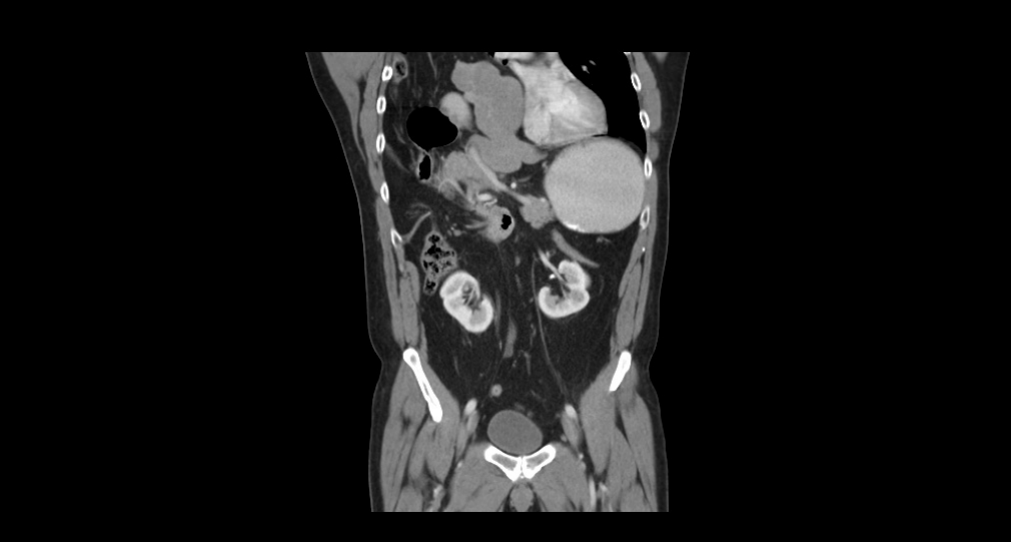
**Coronal CT demonstrating herniation of the liver, bowel and omentum into his right thorax**. Image also demonstrates the absence of any significant diaphragm which is visible laterally on the image.

**Figure 2:**
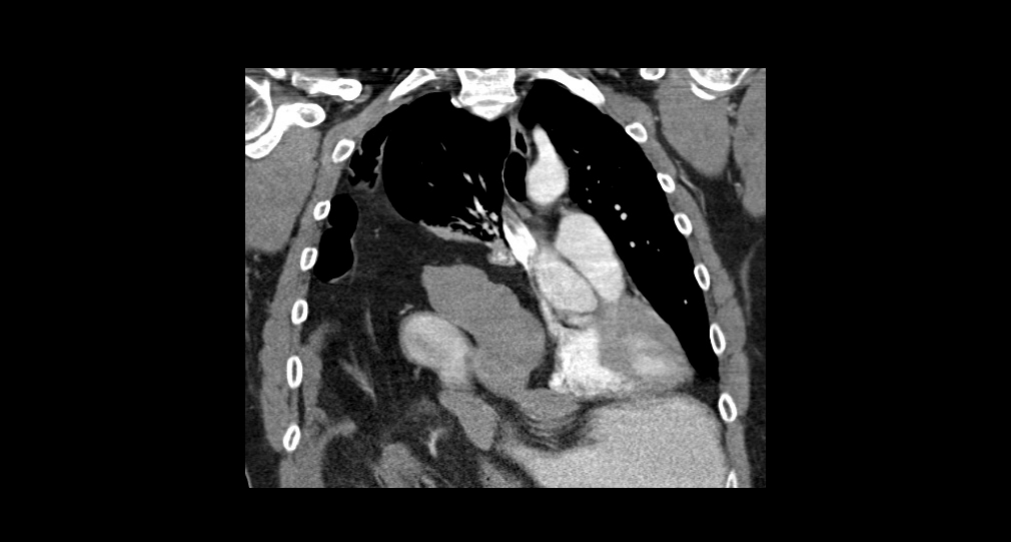
**Coronal CT demonstrating herniated bowel and liver into right thorax**.

**Figure 3:**
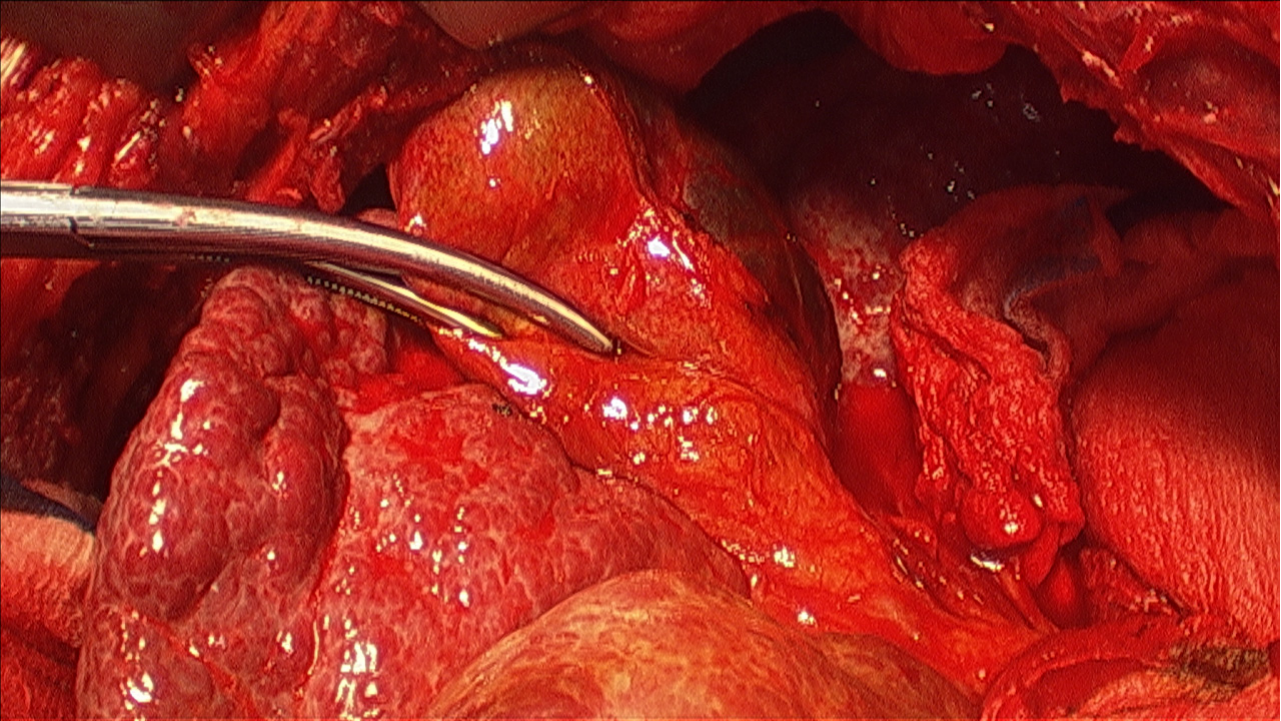
**Intraoperative view**. View as seen through the thoracotomy portion of the incision demonstrating the acutely inflamed gallbladder and macronodular of liver. (Clamp is on the inflamed gallbladder.).

**Figure 4:**
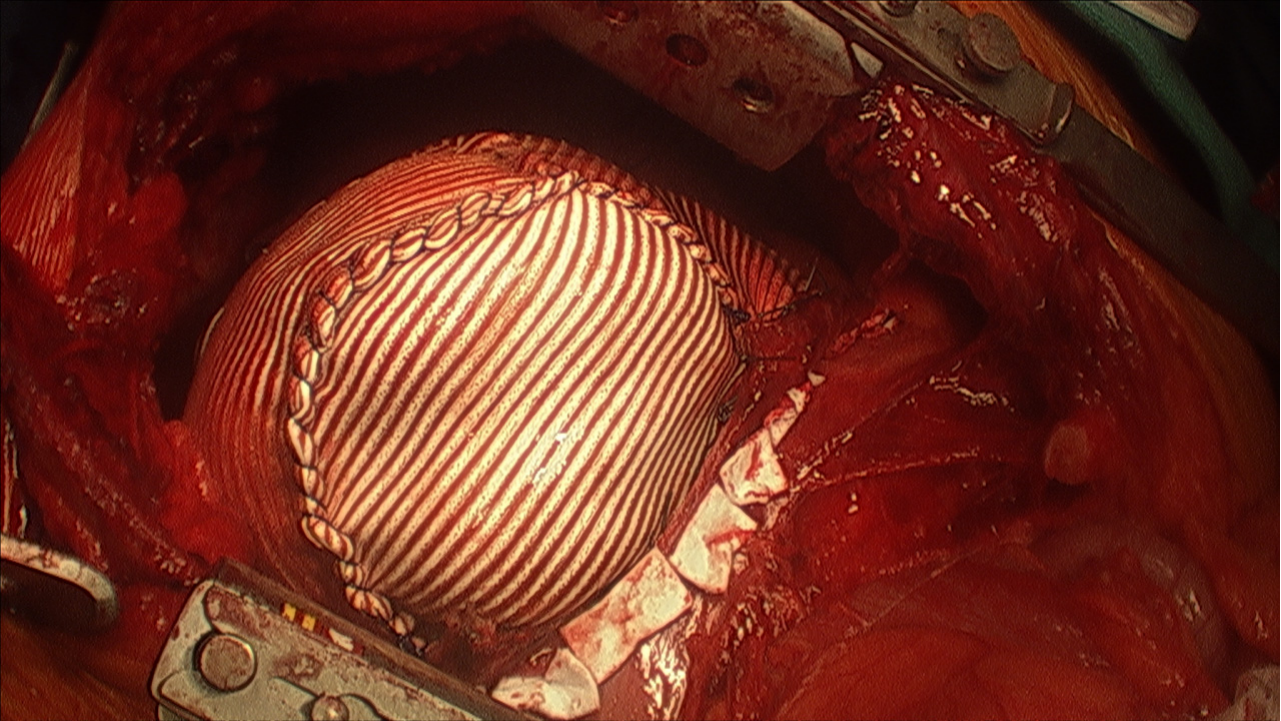
**e PTFE prosthetic patch**. Final prosthetic patch reconstruction covering the dome of his liver after reduction of the liver into the right upper quadrant of his abdomen, as viewed through the thoracoabdominal incision.

**Figure 5:**
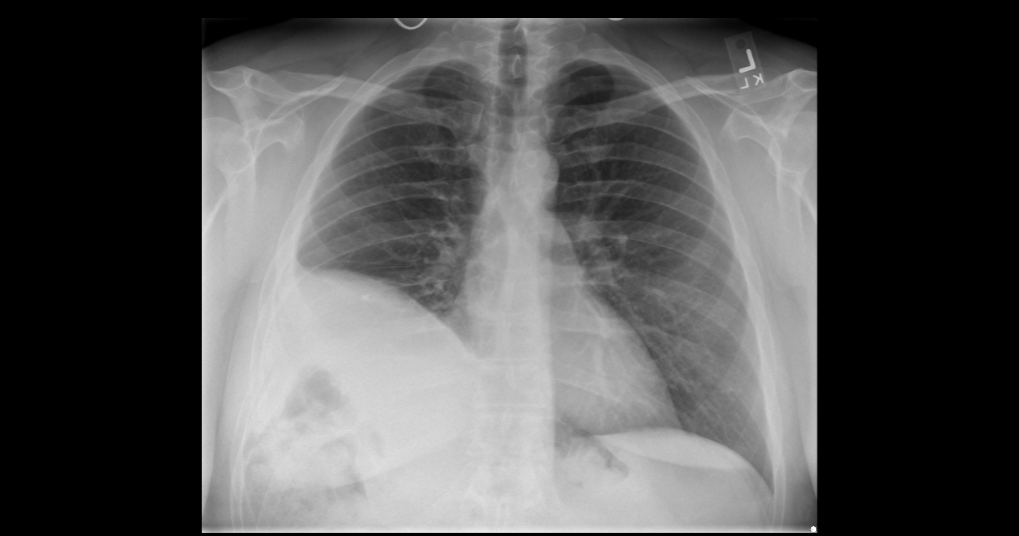
**Chest X-ray following surgery**. Posteroanterior view of his chest one year after repair. His diaphragm is elevated due to the use of prosthetic mesh and the repair is intact without evidence of recurrence of the hernia.

**Figure 6:**
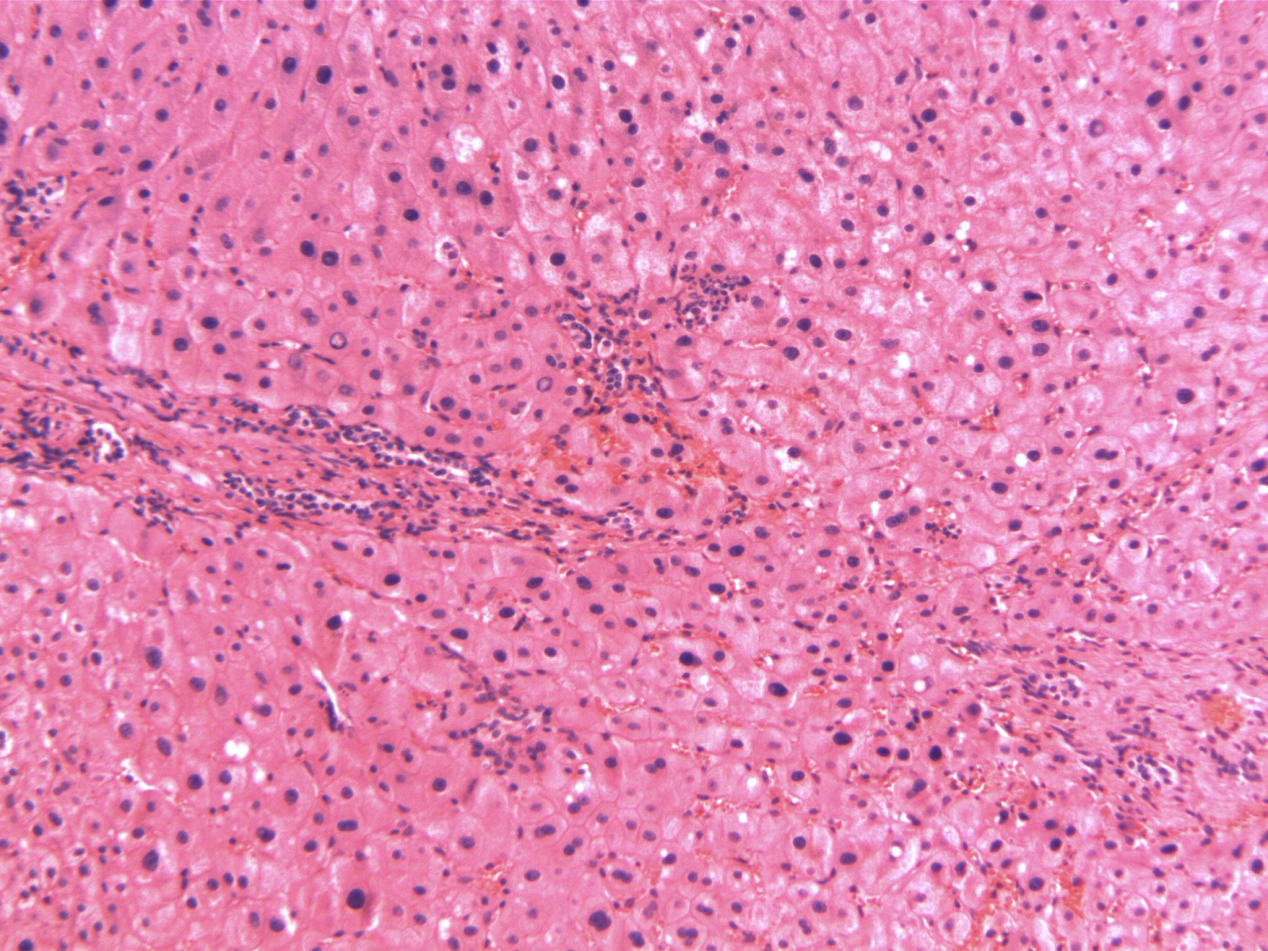
**FNH**. This hematoxylin and eosin stained section of his liver demonstrates the characteristic findings of Focal Nodular Hyperplasia (FNH). The salient finding of the slide includes hyperplastic hepatocytes diffusely distributed without evidence of significant fibrosis.

A 54-year-old Caucasian man presented with epigastric pain associated with nausea without fever or respiratory symptoms. He had a known history of Bochdalek hernia diagnosed two years prior. Medical records indicated that our patient was seen previously by a thoracic surgeon who recommended nonoperative treatment. It was not known if the extent of herniated viscera had worsened in the interim as imaging studies were not available. A physical examination was significant for diminished breath sounds over his right thorax; his abdominal examination was benign. Laboratory data revealed mild leukocytosis with normal serum chemistry and liver-associated enzymes. Radiographic evaluation with axial CT confirmed a massive hernia of Bochdalek with multiple displaced organs into his right thorax (Figure 1 and Figure 2). Initial management included nasogastric decompression and intravenous fluid resuscitation with the presumptive diagnosis of bowel incarceration. After 24 hours, our patient was then taken to the operating theater for repair of the hernia. Given the size of the defect, the surgical approach was a thoracoabdominal incision to allow simultaneous access to both his abdominal and thoracic cavities. At surgery, near complete agenesis of his right hemidiaphragm was noted. Following reduction of his stomach, omentum and bowel, the liver was noted to be grossly abnormal with a macronodular appearance. Further examinationination confirmed acute cholecystitis of the displaced gallbladder (Figure 3). Nearly his entire liver was situated in his lower thorax with its bilio-vascular pedicle stretched as it was pulled into his chest. Following cholecystectomy, a liver biopsy was taken prior to reducing his liver and other organs back into his abdomen. His diaphragm was reconstructed with expanded polytetrafluoroethylene (e-PTFE; 0.2 mm, WL Gore and Assoc., Flagstaff, AZ) (Figure 4). Due to concern for loss of domain and the possible development of abdominal compartment syndrome (ACS), a smaller prosthetic patch was used to close the abdominal fascia to decrease the risk of abdominal hypertension. The right lower lobe of his lung was noted to be hypoplastic; malrotation of the bowel was not observed. Our patient was extubated after surgery and nasogastric decompression was used for 48 hours prior to advancement of oral intake. Postoperatively, our patient was monitored for the development of abdominal hypertension with transurethral bladder pressure measurements for 24 hours [3]. Fortunately, there were no concerns for ACS and our patient had an unremarkable postoperative recovery. At follow-up one year later, our patient noted significant improvement in his activity level as compared to his preoperative state and chest radiography confirmed acceptable separation of his chest and abdominal cavities without recurrence (Figure 5). The liver biopsy returned with the diagnosis of NRH (Figure 6). The pathogenesis of NRH is unknown, but thought to be due to hepatic blood flow disturbances [4].

## Discussion

First reported by Vincent Alexander Bochdalek in 1848, congenital posterolateral hernia of the diaphragm occurs due to persistence of the pleuroperitoneal canal owing to non-fusion of the pleuroperitoneal folds [5]. Fewer than 200 cases of adult diaphragmatic hernia of Bochdalek have been reported and even fewer right-sided hernias in adults [2]. These hernias are often identified incidentally on imaging or can come to medical attention due to complications of the herniated abdominal organs.

This rare case demonstrates two very unusual findings in association with a right-sided diaphragmatic hernia of Bochdalek that have previously not been reported. The first is the development of cholecystitis of the displaced intrathoracic gallbladder. This was the reason our patient came to medical attention and subsequently underwent surgical exploration. It is not possible to attribute causality to the development of cholecystitis with the ectopic location of the gallbladder, however, it is interesting that our patient did have cholecystitis without gallstones. Also of note is that, although our patient's gallbladder was displaced, he experienced epigastric pain and nausea similar to patients without gallbladder displacement. This is likely due to the preservation of the normal splanchnic innervation of the gallbladder despite the abnormal location. Fortunately for this patient, the gallbladder was removed prior to the development of a complication such as perforation or gangrene that could have resulted in empyema thoracis and would have complicated the use of a prosthetic mesh. The second unique finding in this case is NRH of the ectopic liver. This non-cirrhotic liver condition is characterized on histology by diffuse micronodular hyperplasia of the liver cells in the absence of fibrosis [4]. It is believed that nodular regenerative hyperplasia is a secondary and nonspecific tissue adaptation to heterogeneous distribution of blood flow to the liver and does not represent a specific entity [4]. It is conceivable that in this patient, with nearly the entire liver situated in the thorax, the resulting stretch of the vascular pedicle could result in blood flow abnormalities and the possible development of NRH. Clinically, most cases of NRH are silent; however, some patients may have abnormal liver enzymes and rarely progress to portal hypertension. The patient described in this report presented with normal liver enzymes without evidence of hepatic dysfunction or stigmata of end stage liver disease. At one-year follow-up, he had no clinical signs of portal hypertension.

This case also demonstrates several important concepts in terms of surgical management of this complex condition. One possible complication of reduction of large hernias is the loss of abdominal domain and the potential development of intra-abdominal hypertension. Due to the long-standing displacement of various abdominal organs outside the abdominal compartment, there is a decrease in the abdomen's capacity to accommodate the herniated organs when they are reintroduced. Abdominal hypertension can lead to ACS characterized by multiorgan dysfunction [3]. To minimize the risk of development of ACS, prosthetic mesh closure of the abdominal fascia was undertaken to increase the abdominal compliance. A similar strategy has been described in severely injured trauma patients to prevent ACS [6]. During the closure of the abdomen, our patient was monitored for increases in the peak airway pressure, an early sign of ACS [3]. A second caveat is the reconstruction of the defective diaphragm. The use of a prosthetic patch to restore the absent diaphragm is the preferred technique for creating a permanent partition between the two cavities and preventing recurrence. Among patients with large hernias, such as our patient, there is often a deficiency of diaphragmatic tissue to allow primary closure of the defect. Prosthetic materials used in this situation should be nonabsorbable and provide a barrier against air and fluid. This author's preference is 0.2 mm thickness e-PTFE because it is flexible and impervious to air and fluid.

## Conclusion

Hernias of Bochdalek are rare congenital abnormalities that can present with unusual complications related to the ectopic viscera. Massive hernias of the right-side can present with disorders of the liver and gallbladder that are not be seen with hernias of the left side. Potential loss of domain and the possible development of intra-abdominal hypertension should be addressed at the time of repair. Following correction, the patient can expect excellent long term quality of life.

## Abbreviations

ACS: abdominal compartment syndrome; CT: computed tomography: e-PTFE: expanded polytetrafluoroethylene; NRH: nodular regenerative hyperplasia.

## Consent

Written informed consent was obtained from the patient for publication of this case report and any accompanying images. A copy of the written consent is available for review by the Editor-in-Chief of this journal.

## Competing interests

The author declares that they have no competing interests.
